# Cartilage Repair and Subchondral Bone Migration Using 3D Printing Osteochondral Composites: A One-Year-Period Study in Rabbit Trochlea

**DOI:** 10.1155/2014/746138

**Published:** 2014-08-07

**Authors:** Weijie Zhang, Qin Lian, Dichen Li, Kunzheng Wang, Dingjun Hao, Weiguo Bian, Jiankang He, Zhongmin Jin

**Affiliations:** ^1^State Key Laboratory for Manufacturing Systems Engineering, Xi'an Jiaotong University, Xi'an 710054, China; ^2^The First Department of Orthopaedics, The Second Affiliated Hospital, Health Science Center, Xi'an Jiaotong University, Xi'an 710004, China; ^3^Department of Joint Surgery, Hong Hui Hospital, Health Science Center, Xi'an Jiaotong University, Xi'an 710054, China; ^4^Department of Spine Surgery, Hong Hui Hospital, Health Science Center, Xi'an Jiaotong University, Xi'an 710054, China; ^5^Department of Orthopaedics, The First Affiliated Hospital, Health Science Center, Xi'an Jiaotong University, Xi'an 710061, China; ^6^Institute of Medical and Biological Engineering, School of Mechanical Engineering, University of Leeds, Leeds LS2 9JT, UK

## Abstract

Increasing evidences show that subchondral bone may play a significant role in the repair or progression of cartilage damage* in situ*. However, the exact change of subchondral bone during osteochondral repair is still poorly understood. In this paper, biphasic osteochondral composite scaffolds were fabricated by 3D printing technology using PEG hydrogel and *β*-TCP ceramic and then implanted in rabbit trochlea within a critical size defect model. Animals were euthanized at 1, 2, 4, 8, 16, 24, and 52 weeks after implantation. Histological results showed that hyaline-like cartilage formed along with white smooth surface and invisible margin at 24 weeks postoperatively, typical tidemark formation at 52 weeks. The repaired subchondral bone formed from 16 to 52 weeks in a “flow like” manner from surrounding bone to the defect center gradually. Statistical analysis illustrated that both subchondral bone volume and migration area percentage were highly correlated with the gross appearance Wayne score of repaired cartilage. Therefore, subchondral bone migration is related to cartilage repair for critical size osteochondral defects. Furthermore, the subchondral bone remodeling proceeds in a “flow like” manner and repaired cartilage with tidemark implies that the biphasic PEG/*β*-TCP composites fabricated by 3D printing provides a feasible strategy for osteochondral tissue engineering application.

## 1. Introduction

Although cartilage repair has been studied for many years, the regeneration mechanism is still poorly understood, and the quality of repaired cartilage is far from satisfaction and functional restoration [1, 2]. Increasing evidences show that cartilage regeneration is not only tissue engineering trielements related, but other mechanisms (such as oxygen gradient, molecular, and subchondral bone) might have been involved in the process [3–6].

Osteochondral unit has been receiving increased attention in cartilage research [5, 7–9]. As we know, cartilage and bone act in concert to perform a mechanical functional unit, cartilage as a bearing and bone as a structural girder and shock absorber [5]. Meanwhile, there is ample evidence of intensive crosstalk between the articular cartilage and the subchondral bone in synovial joints [10–12]; this interaction is essential for the maintenance of the cartilage-bone unit [11].

Henderson and La Valette [13] observed that the alteration of the subchondral bone plate upward migration and the development of intralesional osteophytes occurred spontaneously in large chondral lesions; likewise, Qiu et al. [14] and Orth et al. [15, 16] independently found subchondral bone plate migration in rabbit spontaneous osteochondral repair model using histomorphometry method.

Therefore subchondral bone plate migration including osteoplasia and remodeling reaction, upward or inward migration, might be a common phenomenon in osteochondral repairing process. The role of subchondral bone should be considered in order to achieve functional restoration during osteochondral repair.

However, it is controversial whether subchondral bone repair is correlated with cartilage restoration during long-term repair period. Chevrier et al. [17–20] pointed out that a higher level of bone remodeling activity might be one of the main factors supporting improved cartilage repair when chitosan-GP/blood implants were applied to marrow-stimulated cartilage lesions at early postsurgical time points (from day 1 to day 56). However, Orth et al. [15] found that articular cartilage repair and subchondral bone reconstitution proceeded at a different pace and the advancement of the subchondral bone plate was not related to the diminished articular cartilage repair in rabbit spontaneous osteochondral repair model over a one-year period. Vasara et al. [21] observed that the subchondral reaction was not related to the cartilage repair process, but the increased stiffness of subchondral bone could thus impair maturation and jeopardize the survival of the repair tissue in the long run [21]. Therefore, the exact change of subchondral bone during osteochondral repair is still poorly understood; in addition, inconsistent outcomes exist in available researches.

As we know, biphasic osteochondral composites have gained increasing interest in osteochondral tissue engineering [22–24]. Poly(ethylene glycol) (PEG) hydrogels have been applied extensively for* in vitro *and* in vivo* cartilage tissue engineering [25–27]; PEG hydrogels could be fabricated by photopolymerization of PEG precursors modified with either acrylate or methacrylate moieties in the presence of photoinitiators [28]. Moreover, due to its high biocompatibility, *β*-tricalciumphosphate (*β*-TCP) ceramic is widely employed for subchondral bone and autologous bone graft [29–34].

Stereolithography (SL) is an accurate and easy-to-use 3D printing technology to fabricate complex structures individually in a manner of layer by layer [28]; hence biologically PEG hydrogels could be directly cured on *β*-TCP scaffolds, forming hydrogel-ceramic osteochondral composites, which would be applied to repair osteochondral defects in our research model.

In this paper, we will quantitatively analyze the migration (remodeling) phenomenon of subchondral bone during large critical size osteochondral repair* in vivo* and their relationship with repaired cartilage in long-term repair period.

## 2. Materials and Methods

### 2.1. Scaffold Design and Fabrication Process

**Figure 1:**
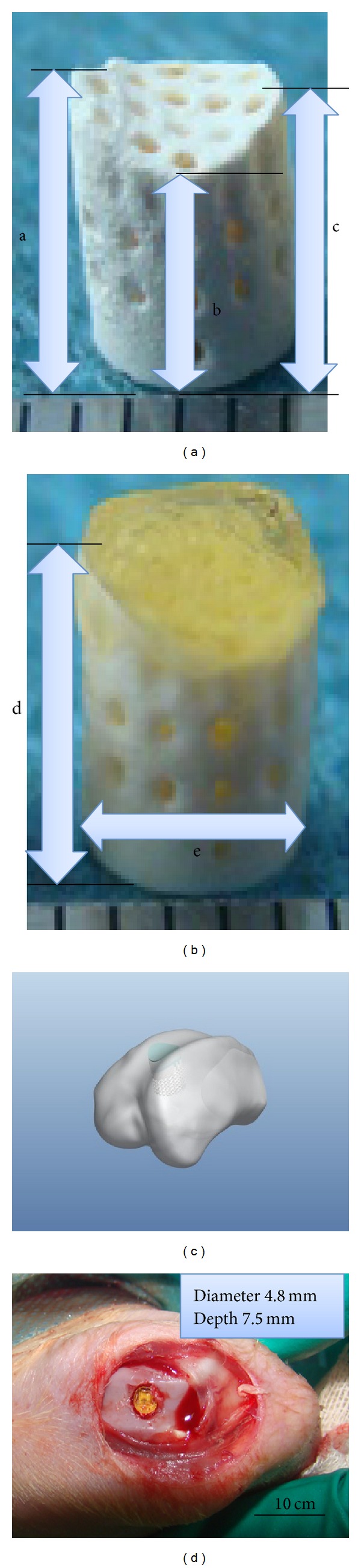
Osteochondral composite and animal experiment: *β*-TCP scaffold (Figure 1(a)), PEG/*β*-TCP osteochondral composite (Figure 1(b)); detailed sizes of composite were as follows: a: 6.5 mm, b: 5.5 mm, c: 6 mm, d: 7 mm, and e: 4.6 mm. Illustration for scaffold implantation in rabbit trochlea (Figure 1(c)) and animal experiment (Figure 1(d)).

Biomimetic osteochondral composite was fabricated by 3D printing technology. Briefly, polyethylene glycol 400 diacrylate (PEG(400)DA, MW = 508, Baoman Biochemistry Co., Ltd.) was prepared as previously described [35]. PEGDA was purified by precipitation in diethyl ether followed by gel filtration chromatography (Sephade G-25) and then dialysed with molecular weight cutoff of 500 Da against deionized H_2_O (Spectrum, Rancho Dominguez, CA) [25].Our previous study showed that PEG hydrogel with desired mechanical property could be prepared via controlling the concentration of PEG(400)DA solution and stereolithography parameters [35]. The *β*-TCP ceramic scaffold was fabricated by gel casting process [36, 37]. Anatomy shaped hydrogel CAD models were input to a custom-made stereolithography machine (SPS150B system, Shaanxi Hengtong Intelligent Machine Co. Ltd., China), and laser power was set on at 100 mW. PEG hydrogels were directly cured on *β*-TCP scaffolds to fabricate biphasic hydrogel-ceramic osteochondral composites (with 4.6 mm in diameter/mm high for PEG/*β*-TCP cylinder and 0.5 mm high for PEG hydrogel part) (as Figures 1(a) and 1(b) showed), with mechanical properties matched cartilage and subchondral bone parts. The compressive strength of PEG hydrogel for cartilage part was 0.75 MPa [35], while *β*-TCP ceramic for bone scaffold was 12.6 ± 0.3 MPa. Other properties for ceramic scaffolds were as follows: 700–900 *µ*m pore size, 200–500 *µ*m interconnected pore size, 50–65% porosity, and fully interconnected [37].

### 2.2. Animal Experiments

Forty male New Zealand white rabbits (6 months skeletal maturity; 3–3.5 kg) used in this study were obtained from experimental animal center of Xi'an Jiaotong University. All animal experiments were approved by the Laboratory Animal Care Committee of Xi'an Jiaotong University, following the Guide for the Care and Use of Laboratory Animals [38]. Briefly, under sterile conditions, engineered implants were then implanted in 35 cylindrical osteochondral defects (diameter 4.8 mm and depth 7.5 mm) created in the right trochlea groove of rabbit knees (as shown in Figures 1(c) and 1(d)); 0.5 mm depth void was left from the cartilage surface after surgery; 5 empty defects with the same size were served as blank controls. For each contralateral knee, sham operation was carried out (with identical wounds in the opposite knee left untreated) as sham controls. Experimental samples of 1, 2, 4, 8, 16, 24, and 52 weeks postoperatively were defined as E1, E2, E4, E8, E16, E24, and E52, respectively, while sham controls were defined as S1, S2, S4, S8, S16, S24, and S52, respectively, blank group at 24 weeks as C24, with 5 samples for each group. Animals were allowed immediate, unrestricted, postoperative activity in individual cages. Animals were euthanized at 1, 2, 4, 8, 16, 24, and 52 weeks postoperatively. The reparative osteochondral tissues were sampled from rabbit distal femurs and then processed for gross appearance assessment, Micro-CT scanning, and histology staining.

### 2.3. Gross Observation

Each sample was evaluated grossly by three independent observers according to Wayne scoring system (available as a supplemental file) (see Supplementary Material available online at http://dx.doi.org/10.1155/2014/746138) [39]. For gross score evaluation, four items were involved including defect coverage, tissue color, defect margin, and surface, with a total score of 16 points.

### 2.4. Sampling and Micro-CT Evaluation

All of samples were scanned using a micro-CT scanner (Inveon Micro-CT, Siemens, Germany). A scan of 360 degrees was carried out at a voltage of 30 kV and a current of 500 *μ*A and an exposure time of 3000 ms. Three-dimensional (3D) reconstructions were created using Mimics software (Materialize, version 13.0, Leuven, Belgium). The reconstructed datasets had a voxel size of 41.76 *μ*m.

**Figure 2:**
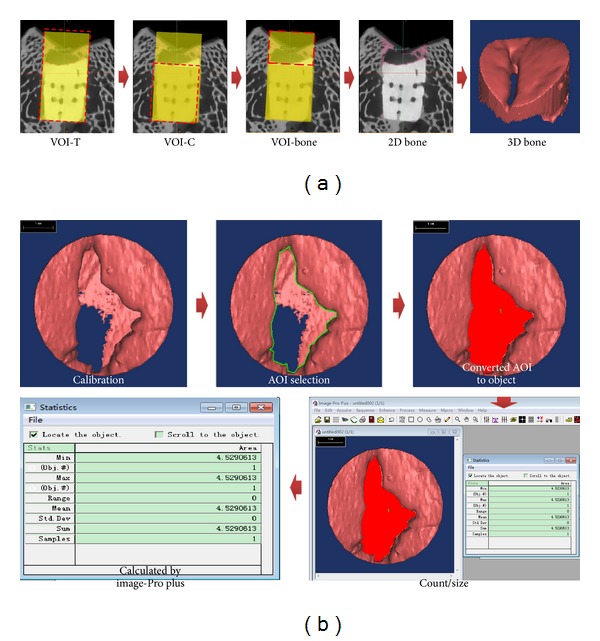
Flow chart of quantitative analysis for repaired subchondral bone: subchondral bone volume (Figure 2(a)) and subchondral bone migration area percentage (Figure 2(b)) (the remaining area percent of the defect apart from the red void one). Calibration→AOI selection→Convert AOI to Object→Count/Size.

The total cylinder volume of interests (VOIs-T) (4.8 mm in diameter and 10 mm in height) including osteochondral composite was selected from the reconstructed datasets. Then, a cylinder ceramic volume of interests (VOIs-C) was established using the same center of the bottom cycle surface with 4.8 mm in diameter and 6mm in height. The repaired subchondral bone VOI (VOI-bone) was obtained by subtraction operation between VOIs-T and VOIs-C. The gray thresholding of subchondral bone was defined in the range of 156 and 1462. The volume of VOI-bone was obtained from Mimics software (Materialize, Leuven, Belgium) (as illustrated in Figure 2(a)); then the subchondral bone volume was obtained.

The subchondral bone migration area was defined as the projection area of the three-dimensional (3D) reconstructed repaired subchondral bone (VOI-bone) on the top view direction. Briefly, the frontier tongue of migrating subchondral bone was recorded on the top view picture of the repaired subchondral bone (VOI-bone); the recorded line then enclosed as an area (projected area from the vertical direction) which represented the remaining area between the repaired subchondral bone which is not occupied.

The projection area picture was calibrated; then the subchondral bone migration area percentage (the remaining area percent of the defect apart from the red remaining area) was selected as AOI (area of interest); selected AOI area was converted to object and processed for count/size calculation using Image-Pro Plus software (Media cybernetics, Inc), which indicated the extent of the defect occupied by the repaired subchondral bone (VOI-bone) (as illustrated in Figure 2(b)).

### 2.5. Histology and Staining

Samples at 16 weeks, 24 weeks, and 52 weeks were processed to histological evaluation. All specimens were fixed in 10% (v/v) buffered formalin for 48 hours; specimens were decalcified in 10% EDTA, dehydrated, embedded in paraffin, and sectioned at 4 *μ*m. Safranin O/Fast green stained sections were merged together from several individual photos in order to get a full view of panorama and then assessed by three independent observers under double blind condition for regenerative changes using Wayne scoring system (available as a supplemental file) [39]. Histology score is composed of matrix points, cell distribution points, smoothness points of the surface, safranin O stain points, and safranin O-stained area points. Subsequently, the full histological score was 19 points. Due to sample preparation process, CaP particles of ceramic were dissolved during the decalcification procedure with empty spots left; PEG hydrogel was also dissolved during dehydration process. The location hydrogel part was marked with red dashed box in stained slices; decalcified ceramic was surrounded by repaired bone tissue (as Figure 4 showed).

### 2.6. Immunological Characterization

Immunohistochemical staining was performed to identify expression of tissue-specific proteins in regenerated tissue according to previously established methods [40]. Type II collagen (COL-II) (anti-collagen-II antibody ab3092, 1 : 100, Abcam, Cambridge, MA, USA) was immunolocalized to identify expression of chondrogenic protein. Type I collagen (COL-I) (anti-collagen-I antibody ab90395, 1 : 150, Abcam, Cambridge, MA, USA) was immunostained to identify whether any cartilage degeneration occurred. Collagen-X immunohistochemical staining (anti-collagen X antibody ab49945, 1 : 200, Abcam, Cambridge, MA, USA) was carried out to identify the mature extent of repaired cartilage.

### 2.7. Biochemical Analysis

Constructs harvested at 24 and 52 weeks were digested with 125 mg/mL papain (Sigma) in 50 mM phosphate buffer supplemented with 2 mM N-acetyl cysteine and 2 mM ethylenediaminetetraacetic acid (EDTA) at 65°C overnight. GAGs was assessed by the Blyscan Glycosaminoglycan Assay Kit (Biocolor) following the manufacturer's instructions; absorbance was measured at 656 nm using a microplate reader. Total collagen content was determined using the Sircol Assay Kit (Biocolor) according to the manufacturer's suggested protocol. Briefly, the samples were digested with 0.1 mg/mL pepsin (Sigma) supplemented with 0.5 M acetic acid (to keep enzyme activity) at 4°C overnight; bovine collagen-I solution (Biocolor) was used as a standard; absorbance was measured at 555 nm.

### 2.8. FTIR of PEG Hydrogel

Before and 52 weeks after implantation, FTIR was applied to evaluate whether the content of PEG hydrogel shows any change during the repairing. Briefly, PEG hydrogel was sampled from the osteochondral repair construct, lyophilized and mixed with potassium bromide, pelletized, and recorded in transmission mode using a FTIR spectrometer (Vetex70, Bruker, Germany) in the 500–4000 cm^−1^ region (4 cm^−1^ resolution, average 64 scans).

### 2.9. Mechanical Property of PEG Hydrogel

Before and 52 weeks after implantation, compressive tests were carried out to evaluate whether mechanical properties of PEG hydrogel show any change during the repairing process. Each sample was tested by static compressive test machine (universal computer-controlled electronic testing machine, type 8503, SANS, Co. Ltd.) at a rate of 0.5 mm/min in room temperature; 5 samples were involved before and 52 weeks after implantation, respectively.

### 2.10. Statistics Analysis

All data were presented as means ± standard deviations (s.d.). To test the significance of observed differences between study groups, statistical analyses were performed using* t*-test (for 2 groups comparison) and one-way ANOVA with Tukey post-hoc test (for groups more than two), respectively, only when homogeneity of variance was achieved. Pearson correlation coefficients were calculated for comparison of the changes in subchondral bone and cartilage characteristics, time dependent characterization of subchondral bone. Then curve fit estimation was carried out to validate the regression model when significant correlation was fulfilled. A level of *P* < 0.05 was considered statistically significant. All statistical analyses were performed with the SPSS software package (version 17.0, SPSS Inc., Chicago, IL).

## 3. Results

### 3.1. Gross Appearance and Quantitative Scoring

In this study, sham operation was carried out on each contralateral knee, (with identical wounds in the opposite knee left untreated) as sham controls, defined as S1, S2, S4, S8, S16, S24, and S52, respectively. Because the gross appearance and histology results appeared similar to each other, we chose S24 as representative sham control in this paper.

**Figure 3:**
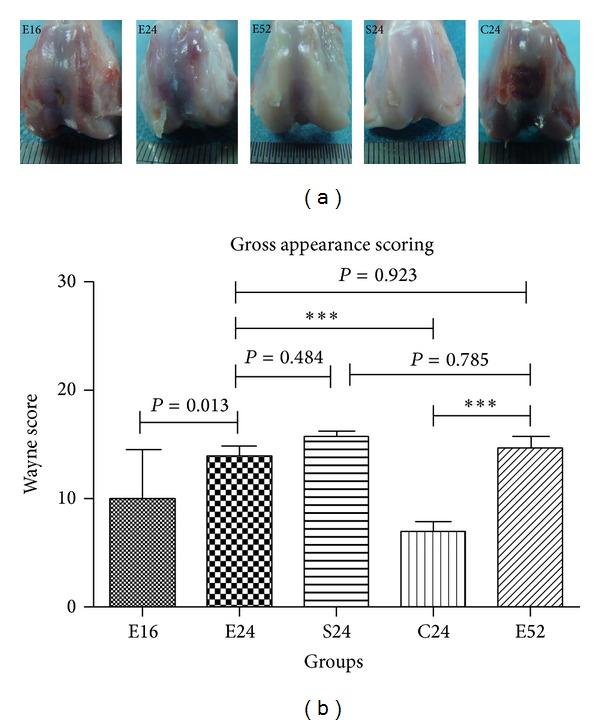
Gross appearance and Wayne score of repaired cartilage (***P < 0.001).

No obvious immunological or infectious complications were observed throughout the experiment. At 16 weeks, defects treated with PEG/*β*-TCP osteochondral composite were filled with repaired white opaque tissue with more than 75 percentage void area; the surface was relatively rough compared with sham operation groups; most of margins were still visible (Figure 3(a)). At 24 weeks, defects were mainly fulfilled with cartilage-like tissue, with smooth but raised surface and 50% of the margin were invisible; the color of repaired tissue was close to that of normal one (Figure 3(a)). At 52 weeks, the repaired tissue owned hyaline-like characterization, such as white normal color, smooth surface comparable with sham groups; the margin was nearly invisible (Figure 3(a)), while untreated defects were insufficiently filled with red fibrous tissue, with rough surface and entire visible defect margin (Figure 3(a)).

The quality of cartilage repair tissue was graded using Wayne score system [39]. At 16 weeks, cartilage repair was relatively incomplete (with total average score 10.00 ± 4.472, Figure 3(b)). By 24 weeks postoperatively, cartilage repair was significantly improved (13.83 ± 0.983, *P* = 0.013, Figure 3(b)), compared with the one at 16 weeks. No obvious improvement was observed at 52 weeks for gross appearance (E24 VS.E52, *P* = 0.923, Figure 3(b)); however, gross score for 52 weeks showed no obvious difference with that of sham operation groups at 24 weeks (*P* = 0.785, Figure 3(b)). Respectively, tissue repair at these three time points showed significant difference with the blank control groups at 24 weeks (*P* < 0.001, Figure 3(b)), suggesting that biphasic PEG/*β*-TCP osteochondral composite enhances osteochondral repair in critical size defect model.

### 3.2. Histological and Immunohistochemical Characterization of Repaired Cartilage

**Figure 4:**
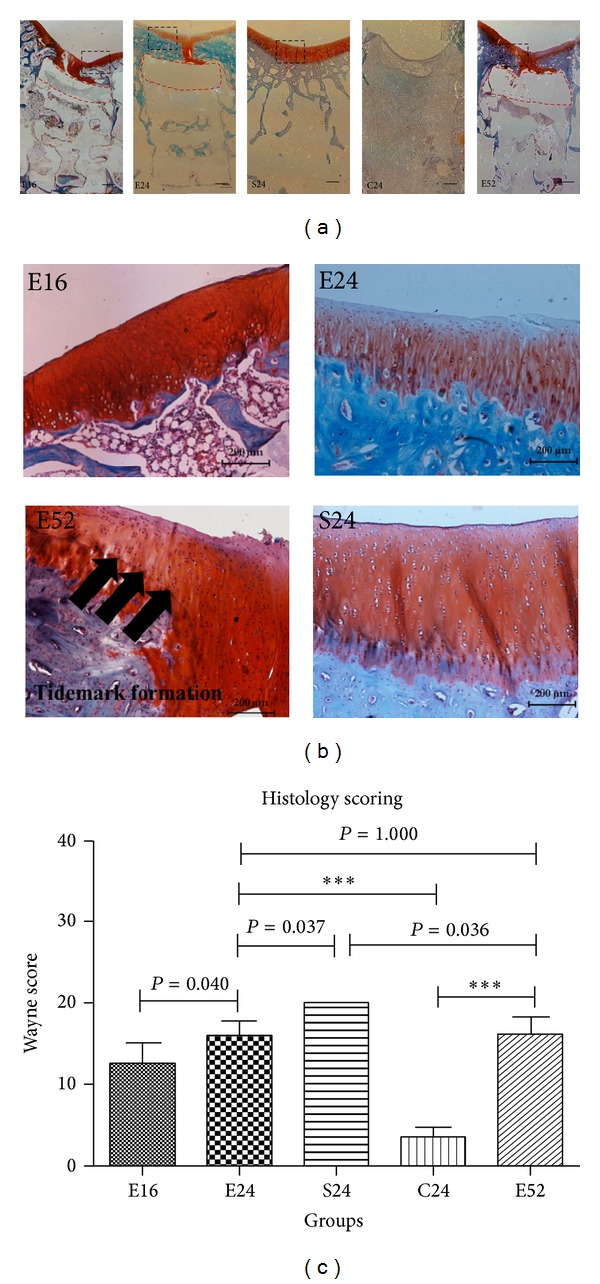
Histology and Wayne score of repaired cartilage: pictures in Figure 4(a) were merged together from several individual photos in order to get a full view of panorama, bar = 500 *μ*m; pictures in Figure 4(b) were magnification of the part in dotted boxes of Figure 4(a), bar = 200 *μ*m; tidemark formation was presented in Figure 4(b), (as blank arrows showed); Figure 4(c) showed comparison of Wayne score in each group, (***P < 0.001). Due to sample preparation process, CaP particles of ceramic were dissolved during the decalcification procedure with empty spots left; PEG hydrogel was also dissolved during dehydration process. The location of hydrogel part was marked with red dashed box in stained slices; decalcified ceramic was surrounded by repaired bone tissue.

At 16 weeks* in vivo*, cartilage repair was uncompleted (total average score 12.67 ± 2.338, Figure 4(a)), the cartilage repaired was hyaline and fibrocartilage mixed, cell organization was irregular with mixed/columnar clusters, and clefts were observed in some cases; at 24 weeks, cartilage repair was improved compared with that of 16 weeks (15.889 ± 1.883, *P* = 0.040, Figure 4(c)), cell distribution became more organized, columnar structure appeared, cartilage surface was as smooth as normal level, and the tidemark was rarely observed; at 52 weeks, although the total histology score was not significantly improved compared with that of 24 weeks (*P* = 1.000, Figure 4(c)), impressively, the tidemark, one of indicators for cartilage maturation, was observed traversing through the critical size defect (as black arrows showed in Figure 4(b)). While, the blank controls were filled with fibro-like tissue, with rare Safranin O stained, no chondrocytic cells were observed in defects (3.667 ± 1.155, Figure 4(c)). Cartilage repair at 16–52 time points were significantly improved compared with blank controls (C24) (*P* < 0.001, Figure 4(c)).

**Figure 5:**
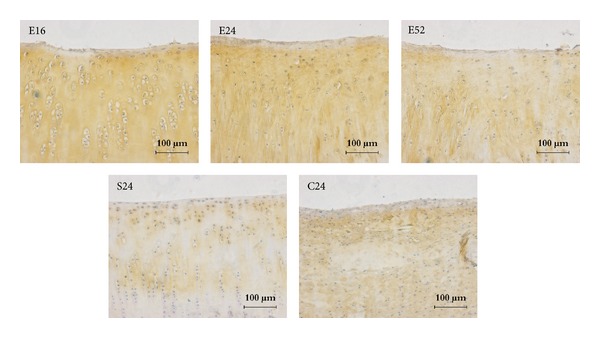
Collagen-II immunohistochemical staining of repaired cartilage. Collagen-II staining in 16, 24, and 52 weeks was intensively expressed, which is comparable to sham control group, while tissue sections at 16 weeks showed limited expression, bar = 100 *μ*m.

**Figure 6:**
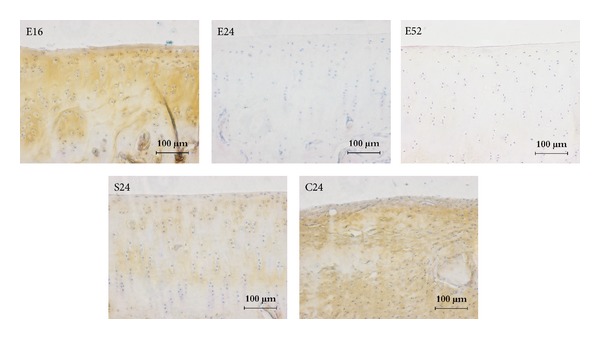
Collagen-I immunohistochemical staining of repaired cartilage. Collagen-I staining was faintly positive at 24 and 52 weeks, which was comparable with sham control group, while tissue sections at 16 weeks were positively stained with collagen I, bar = 100 *μ*m.

**Figure 7:**
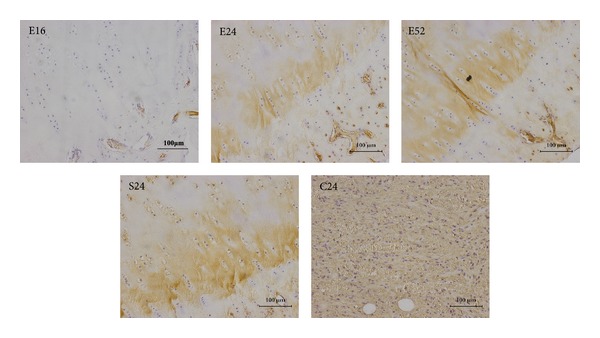
Collagen X immunohistochemical staining of repaired cartilage. Collagen type X staining is more pronounced in 24 and 52 weeks when compared to 16 weeks tissue sections, bar = 100 *μ*m.

Collagen type II staining was strongly positive in 16, 24, and 52 weeks tissue sections. Staining of collagen type II was comparable with that of sham control group (Figure 5), while tissue sections at 16 weeks showed limited expression; collagen-I staining was faintly positive at 24 and 52 weeks, which was comparable with sham control group, while tissue sections at 16 weeks were positively stained with collagen-I (as Figure 6 showed); collagen type X staining is more pronounced in 24 and 52 weeks when compared to 16 weeks tissue sections. No specific staining was observed at blank control group (as Figure 7 showed).

### 3.3. Biochemical Characterization of Repaired Cartilage

**Figure 8:**
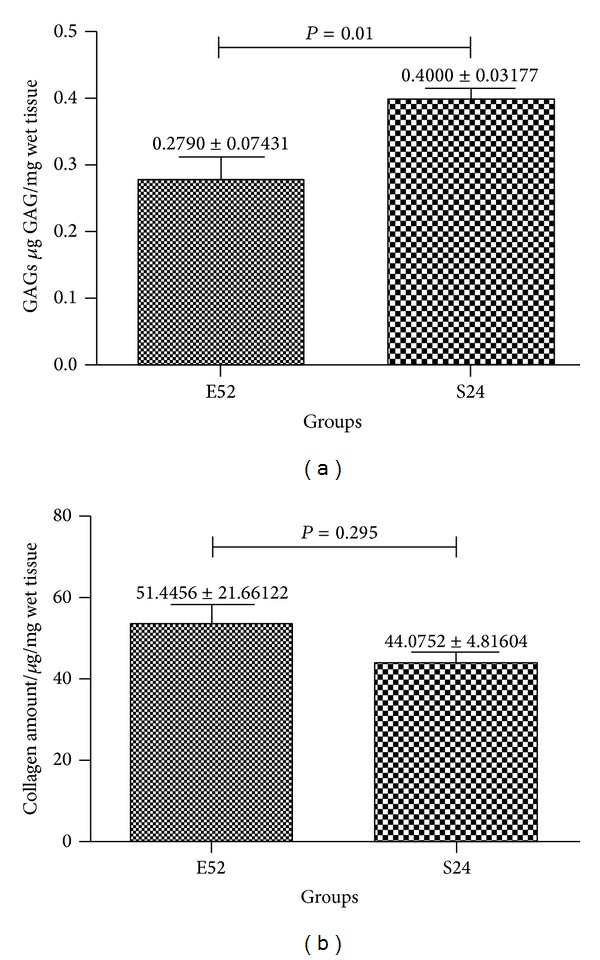
Biochemical characterization of repaired cartilage.

Biochemical results showed that GAGs content at 52 weeks was close to that of sham group, while collagen content was not significantly different with sham group (as Figure 8 showed).

### 3.4. Subchondral Bone Migration Phenomenon during Osteochondral Repair

**Figure 9:**
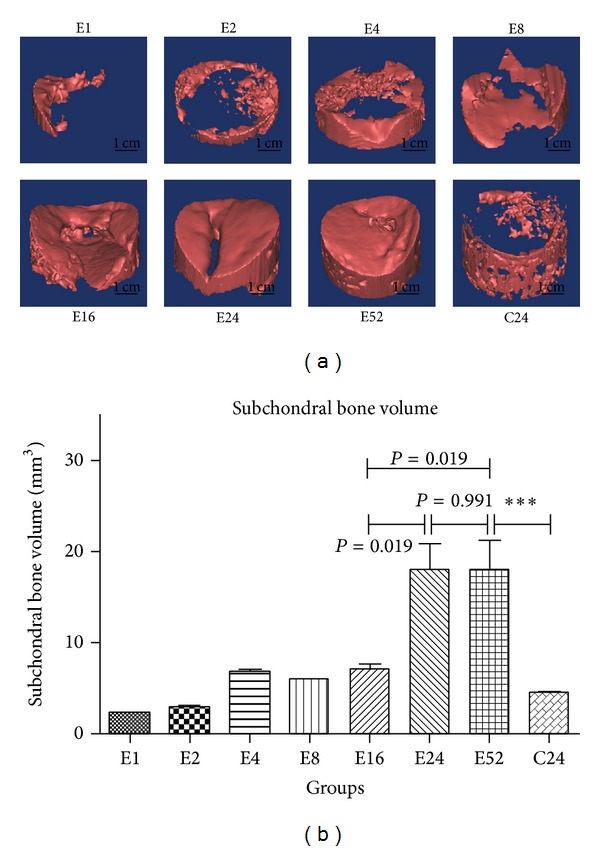
Variation of subchondral bone volume during osteochondral repairing: 3D model of subchondral bone repaired (Figure 9(a)) and statistical results (Figure 9(b)) (***P < 0.001).

The repaired subchondral bone volume was characterized as the part of subchondral bone repaired above osteochondral composite (as Figure 2(a) showed). Between 1 week and 24 weeks, the subchondral bone volume progressively increased from 2.200 mm^3^ to 17.97 mm^3^, (Figure 9(b)), the repaired subchondral bone advanced persistently towards the center of the defect (shown in Figure 9(a)), and the defect margin was nearly joined together at 24 weeks and 52 weeks. The repaired bone volume at 24 weeks was significantly increased compared with that of 16 weeks (*P* = 0.019, Figure 9(b)); no distinct increasement was observed at 52 weeks in contrast with 24 weeks (*P* = 0.991, Figure 9(b)). The amount of subchondral bone at 24 weeks and 52 weeks was significantly larger than that of blank control groups at 24 weeks (C24) (*P* < 0.001, Figure 9(b)).

**Figure 10:**
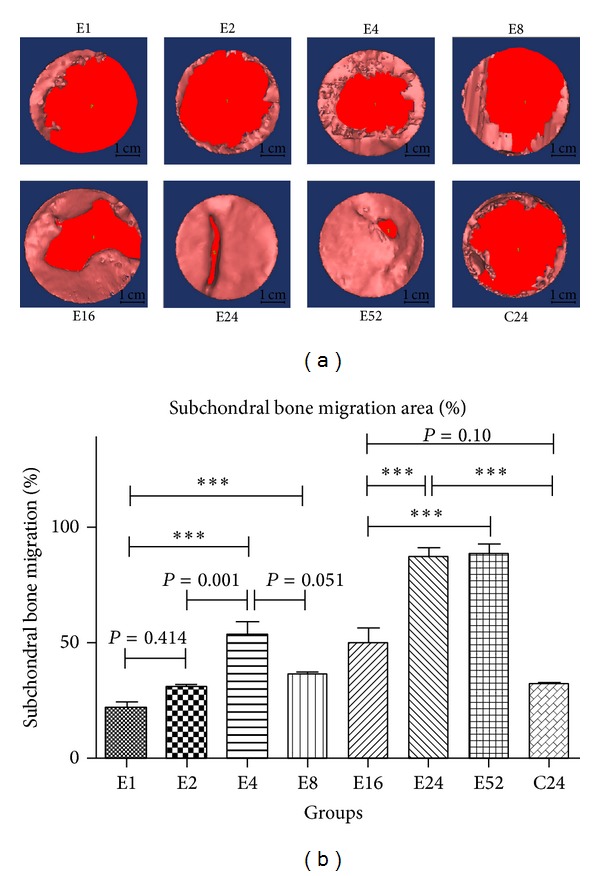
Variation of subchondral bone migration area percent during osteochondral repairing: subchondral bone migration area percentage (Figure 10(a)) and statistical results (Figure 10(b)) (***P < 0.001).

The subchondral bone migration area was defined as the projection area of the subchondral bone on the top view direction; then the area percentage was calculated to confirm to what extent the defect was occupied by the repaired subchondral bone. It could be found that, at the early repair period (1–4 weeks), the migration area percentage increased rapidly approaching to 53.33% at 4 weeks after surgery, (Figure 10(b)); then the migrated subchondral bone exhibited hesitance behavior with a decline in migration area percentage at 8 weeks, although no significant decrease was observed (*P* = 0.051). It reaches to a plateau stage at 24 weeks later, with no obvious increase at 52 weeks (E24 versus E16, *P* = 0.019 and E24 versus E52, *P* = 0.991, Figure 10(b)).

Regarding the subchondral bone migration area, the repaired subchondral bone followed a potential discipline to which the repaired subchondral bone migrated from surrounding bone part to the defect center gradually.

Both subchondral bone volume and subchondral bone migration area percentage were highly correlated with time points in univariate regressions, with *r* ^2^ (coefficient of determination) linear of 0.638, *r* ^2^ quadratic of 0.731, and *r* ^2^ cubic of 0.796 all *P* < 0.001, for regression between subchondral bone volume and time points (as shown in Table 1), likewise with *r* ^2^ (coefficient of determination) linear of 0.639, *r* ^2^ quadratic of 0.761, *r* ^2^ cubic of 0.800, all *P* < 0.001, for regression between subchondral bone migration area percentage and time points (as shown in Table 1).

### 3.5. Relationship between Repaired Cartilage and Subchondral Bone Migration

In order to confirm whether subchondral bone repair would enhance cartilage regeneration with respect to the critical size osteochondral defect repair model, individual changes in subchondral bone volume and subchondral bone migration area percentage were correlated with the gross appearance Wayne score and histology Wayne score. As shown in Table 2, subchondral bone volume showed positive correlation with the gross appearance Wayne score (Pearson's *r* = 0.865, *P* = 0.001); meanwhile, subchondral bone migration area percentage showed similar correlation with the gross appearance Wayne score (Pearson's *r* = 0.923, *P* < 0.001), while, the two quantitative indexes for repaired subchondral bone were not correlated with histology Wayne score (*P* > 0.05).

### 3.6. PEG Hydrogel Characterization during Cartilage Repair

**Figure 11:**
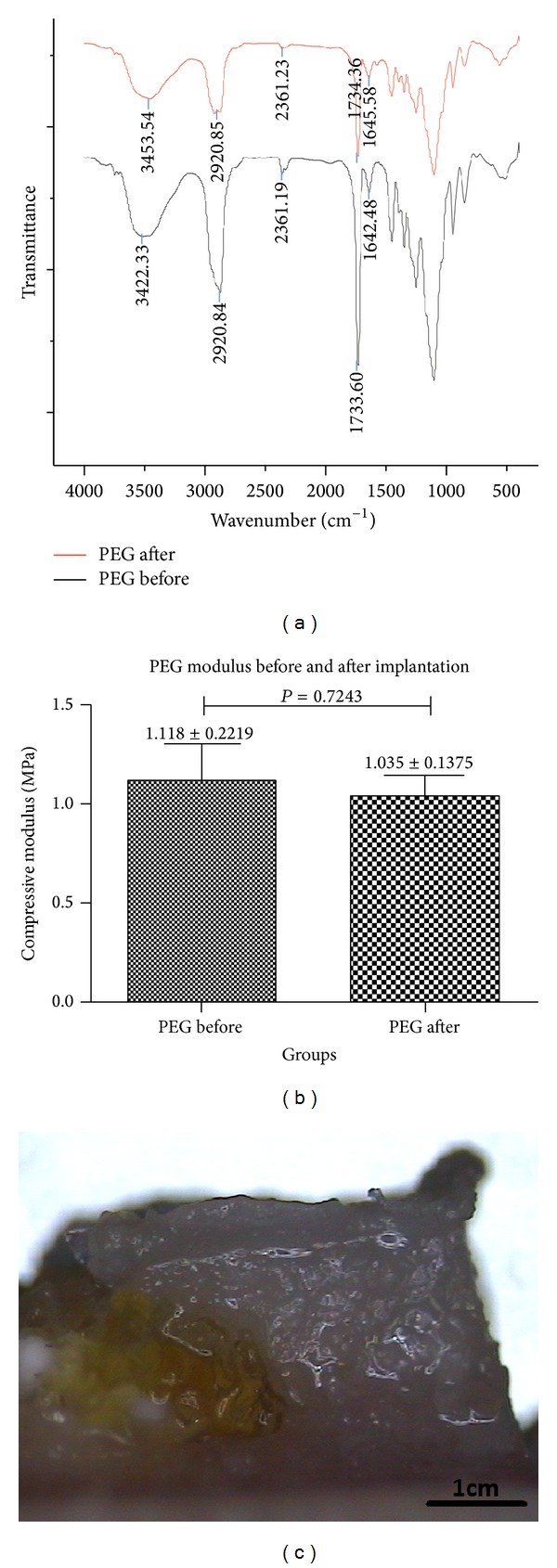
PEG hydrogel analysis before and 52 weeks after implantation. (a) Fourier transform infrared spectroscopy (FTIR) spectra, (b) compressive modulus, and (c) residual PEG hydrogel (as yellow part implied) in regenerated osteochondral plug 52 weeks after implantation.

The implanted PEG hydrogel stayed* in situ* in the whole repair process (as marked with red dashed box in Figure 4, yellow part implied in Figure 11(c)). The FTIR spectra of the PEG hydrogel before and 52 weeks after implantation were shown in Figure 11(a). The peaks centered at 1640 cm^−1^ are attributed to the double bonds of the acrylates (Figure 11(a)), suggesting that most of the −C=C− bonds had been polymerized. The peaks at 1730 cm^−1^ were assigned to the C=O stretching vibration. The absorbance at 3453 cm^−1^ is assigned to the N–H stretching band. The typical peaks location of 52 weeks PEG hydrogel was similar to PEG hydrogel before implantation; the mechanical property of 52 weeks PEG hydrogel was stable compared with that of the initial ones (as Figure 11(b) illustrated).

## 4. Discussion

In this study, there were three major findings: (1) subchondral bone migration is related to cartilage repair for critical size osteochondral defects in one-year period* in vivo*, (2) the subchondral bone remodeling during critical osteochondral repair proceeds with a “flow like” manner, and (3) the biphasic PEG/*β*-TCP composites fabricated by 3D printing provides a feasible strategy for osteochondral tissue engineering application.

### 4.1. Repaired Cartilage in Osteochondral Defect Repair

Recently, biphasic osteochondral composite has gained increasing interest. Matching scaffold with native cartilage compressive properties seems to be crucial for scaffold design [2, 41, 42]. The mechanical support provided by the scaffold is crucial for cellular development and the subsequent excretion of the critical extracellular matrix. Biphasic hydrogel-ceramic osteochondral composites with matched mechanical properties were successfully fabricated by 3D printing technology and then implanted in right trochlea critical size defect (4.8 mm in diameter and 7.5 mm in depth) of skeletal mature NZW rabbit model.

Articular cartilage repair was significantly improved over a one-year period; the repaired tissue showed hyaline-like characterization with white smooth surface, invisible margin at 24 weeks postoperatively; tidemark formed at 52 weeks, reflecting progressive repair process of short, medium, and long-term period in rabbit critical size osteochondral defect model. The repaired cartilage shifted from hyaline/fibrocartilage to mainly hyaline cartilage at 52 weeks, as evidenced by white hyaline-like gross appearance, invisible defect margin (Figure 3(b)), strong safranin O (Figures 4(a) and 4(b)), cell organization (Figure 4(b)), tidemark formation (Figure 4(b)), calcified cartilage formation at 24 and 52 weeks (Figure 7), intensively positive for collagen II and faintly positive for Collagen I (Figures 5 and 6), and intense subchondral bone regeneration. As markers for the quality of repaired cartilage, the Wayne score for gross appearance and histology were both comparable with those of sham operation groups (as Figure 4(c) showed) and significantly improved compared with those of blank control groups (*P* < 0.001).

The tidemark, the basophilic line on articular cartilage sections separating uncalcified cartilage from calcified one, which was one of the representative markers for cartilage maturation [43], was clearly detectable in the repaired cartilage section at 52 weeks. Calcified cartilage below the tidemark forms an interface between the uncalcified cartilage and subchondral bone expresses collagen type X [44]. Calcified cartilage zone is important for suggesting successful osteochondral defect repair. Additional Collagen X immunohistochemical staining was carried out for repaired cartilage. As shown in Figure 7, collagen type X staining is more pronounced in 24 and 52 weeks when compared to 16 weeks tissue sections. Immunohistochemical results demonstrated that the repaired neocartilage at 24 weeks and 52 weeks was positive for collagen X, suggesting that the reparative tissue showed hyaline cartilage characters; these data indicate that biomimetic biphasic PEG/*β*-TCP composites fabricated by 3D printing provide a feasible strategy for osteochondral tissue engineering application.

All these experimental results for cartilage repair suggested that biphasic PEG/*β*-TCP composite fabricated by 3D printing could not only promote the restoration of critical size osteochondral defect in rabbit model, but also enhance the maturation of the repaired cartilage; thus could provide a feasible strategy for osteochondral tissue engineering application.

### 4.2. Time Dependent Subchondral Bone Migration

Cartilage repair is a complex process that takes place over a long period of time. As cartilage-bone unit has been gaining more and more attentions [5, 11], the notion of cartilage repair should turn to the more comprehensive view of osteochondral repair [8]. Increasing evidences showed that the subchondral bone may play a significant role in the onset, repair or progression of cartilage damage [5, 13, 21–26]; thus the role of subchondral bone should be considered in order to achieve functional restoration during osteochondral repair.

The present study revealed that the subchondral bone remodeling within osteochondral defect proceeded following a defined “flow like” manner, by which the repaired subchondral bone migrated from surrounding bone part to the center gradually; the defect margin was nearly joined together at 24 weeks and 52 weeks. The regeneration phenomenon was confirmed by both subchondral bone volume and subchondral bone migration area percentage; moreover, the phenomenon was time dependent and cubic regression showed considerable firm relationship between time points and subchondral bone repair. Identical to the present study, Orth et al. [15] also observed that subchondral bone reconstitution proceeded in a definite chronological order using uncritical size spontaneous repair model, the main difference was the defect model applied; it could be concluded that subchondral bone regeneration phenomenon was time dependent in both spontaneous repair model and critical size defect repair model.

### 4.3. Relationship between Subchondral Bone Migration and Repaired Cartilage

As more and more evidences showed that the subchondral bone may play a significant role during repair or progression of cartilage damage* in situ* [5, 9, 15, 16, 45–48], the role of subchondral bone should be considered in order to achieve functional restoration during osteochondral repair. However, the exact change of subchondral bone during osteochondral repair is still poorly understood; in addition, inconsistent outcomes exist in available researches [15, 17, 18, 21].

Orth et al. [15] found that articular cartilage repair and subchondral bone reconstitution proceed at a different pace and that the advancement of the subchondral bone plate was not related to the diminished articular cartilage repair in a rabbit model of spontaneous osteochondral repair over a one-year period. Vasara et al. observed that the subchondral reaction was not related to the repair process [21]. It could be apparently observed that osteochondral defect models by Orth et al. and Vasara et al. were not critical size defined; the former one was 3.2 mm in diameter in rabbit model [15], while the latter one was 6 mm diameter lesion in goat model which was confirmed to prone to heal spontaneously, with bone filling the base and fibrocartilage filling the area above [49]. It could be speculative that the subchondral remodeling might not be related to the cartilage repair process in spontaneous cartilage repair model.

However, it is controversial whether subchondral bone repair is correlated with cartilage restoration during long-term repair period.

Chevrier et al. [17–20] showed that a higher level of bone remodeling activity is one of the main factors supporting improved cartilage repair when chitosan-GP/blood implants are applied to marrow-stimulated cartilage lesions at early postsurgical time points (from day 1 to day 56), but no further evidence was available for long-term repair process.

In the present study, critical size defects and 3D printing fabricated biphasic osteochondral composites with matched mechanical properties were applied to elucidate the correlation between subchondral bone remodeling and cartilage repair; both subchondral bone volume and subchondral bone migration area percentage were highly correlated with the cartilage gross appearance score (*P* < 0.01), suggesting that the subchondral bone repair was correlated with the cartilage regeneration in critical size defect repair model in a long lasting one-year period.

### 4.4. The Role of PEG Hydrogel during Osteochondral Repair

Due to sample preparation process, CaP particles of ceramic were dissolved during the decalcification procedure with empty spots left; PEG hydrogel was also dissolved during dehydration process. The location hydrogel part was marked with red dashed box in stained slices, while decalcified ceramic was surrounded by repaired bone tissue (as Figure 4 showed).

The implanted PEG hydrogel stayed* in situ* in the whole repair process (as marked with red dashed box in Figure 4, yellow part implied in Figure 11(c)); additionally, the compressive modulus and FTIR of PEG hydrogel were investigated before and 52 weeks after composite scaffold implantation. The mechanical property of implanted PEG hydrogel was stable compared with that of the initial ones (as Figure 11(b) illustrated); meanwhile, the FTIR results also showed that the hydrogel ingredient did not change during the whole repairing period (as Figure 11(a) showed).

Biomechanical microenvironment is known to be important for chondrogenic differentiation of mesenchymal stem cells and matrix production [50, 51]. Moreover, a crucial role is played by the mechanical properties of the tissue engineered cartilage during the healing process, which, ideally, must match those of native cartilage [52]. Matching scaffold and native cartilage compressive properties seems to be crucial for osteochondral scaffold design [2, 41, 42]. Simultaneous delivery of tissue-specific stimuli will provide the most attractive means of tissue regeneration [53]. However, spatial and temporal control of the mechanical properties of single tissue-specific constructs has proved challenging [42]. Likewise, to accurately control contour of osteochondral composites is also an important issue.

Our previous study showed that PEG hydrogel with desired mechanical property could be prepared via controlling the concentration of PEG(400)DA solution and stereolithography parameters [35]

PEG hydrogel might provide biomimetic mechanical environment for osteochondral repair. However, more extensive designed study was needed to illustrate the extent that biomimetic biomechanical environment would impact on the osteochondral repair; for instance, PEG scaffold with different mechanical properties might be applied in osteochondral defect repair* in vivo* in the near future to investigate the detailed effect of biomechanical environment.

### 4.5. Osteochondral Repair Mechanism

Traditional techniques for cartilage repair include marrow stimulation, allografts, and autografts. Although successful in some aspects, each of these techniques has limitations. Despite favorable clinical results, unexplained graft failures sometimes occur. Several unanswered questions remain regarding the biological mechanism of the repair process [21]. For example, factors and modulators affecting the repair process are not known; the maturation process of the repair tissue, organization of the matrix components with time, and the role of subchondral bone in osteochondral repair are not yet well characterized [21]. Increasing evidence shows that osteochondral regeneration is not just tissue engineering trielements derived; other mechanisms (such as oxygen gradient, molecular, and subchondral bone) might have involved in the same process [3–6].

To adequately treat lesions that extend into the subchondral bone, a comprehensive understanding of the regeneration phenomenon of subchondral bone during cartilage repair is necessary. Meanwhile, a profound understanding of the mechanism of osteochondral repair could be critical to develop efficient and effective therapeutic strategies to treat osteochondral defects.

Knee is thought to be a largely mechanically-driven organ. Pertinent to this, bone is a dynamic tissue that adapts to loads by remodeling to meet its mechanical demands (Wolff's law) [54]. Increasing evidences show that bone exhibits a high level of innate repair capability; hence, bone tissue, rather than cartilage, has seen more development as a target for regeneration [2].

The subchondral bone migration phenomenon observed in the present study provides enlightenment for the mechanism of osteochondral repair.* In situ* remodeling or subchondral bone migration seems to enhance the cartilage repair.

The potential mechanism might proceed as follows: with the assessment of bone marrow, the osteochondral defect is spontaneously filled with a blood clot, forming an intermediate tissue with fibrin as scaffold and multiple mesenchymal cells, which might differentiate under the influence of growth factors of (released from platelet) into chondrocytes and osteoblasts that later form the cartilaginous repair tissue and the new subchondral bone [5]. It is well known that mechanical force plays significant role in MSC differentiation and mechanoregulation of skeletogenesis [55, 56]. Under the mechanical environment of knee joint, the subchondral bone is induced to migrate from surrounding bone part into the defect center (as illustrated in Figures 4, 5, 6, and 7, as evidenced by the cartilage tissue underlying the migrated subchondral bone), which in turn becomes maturation gradually. The maturing subchondral bone seems to be crucial for supporting and protecting of new articular cartilage formation.

**Figure 12:**
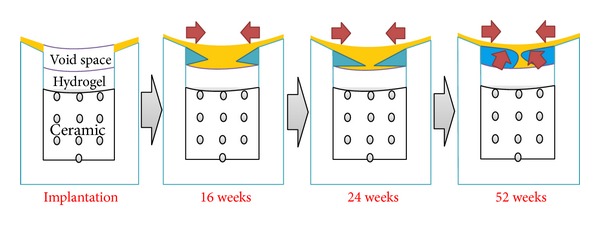
Illustration of subchondral bone migration pattern during critical size defect restoration. The subchondral bone remodeling during critical osteochondral repair proceeds with a “flow like” discipline, by which the repaired subchondral bone migrates from surrounding bone part to the defect center; the defect margin becomes almost completely joined together with each other at 24 weeks and 52 weeks. The yellow part indicates the repaired cartilage, while blue part shows how subchondral bone migrates from periphery to center area and the remodeling process of subchondral bone shape.

Remodeling in subchondral bone observed in present study could be a potential way to enhance cartilage repair through bridge connection (as Figure 12 showed), following a defined “flow like” discipline, by which neighboring cartilage could be able to migrate across the defect, with the aid of migrated subchondral bone bridge to enhance cartilage regeneration in critical size defects. Meanwhile, the migrated subchondral bone become strong enough to provide appropriate support for the maturation of repaired cartilage; this in turn would enhance the cartilage repair outcome. Furthermore, defect wall bone resorption or collapse that occurred in critical size defect could be avoided. Secondary changes in the surrounding bone and articular cartilage may be prevented by restoration of the subchondral bone [5]. Moreover, the lateral integration obstacle could be solved simultaneously (Figures 4(a), 4(b), and 12) which was thought to be major stumbling block to achieve permanent cartilage replacement. Then, we might be able to speculate that osteochondral repair may start from bone; however, more research is required to fully characterize the detailed mechanism of cartilage regeneration.

We recognize one limitation of this study that we did not perform biomechanical evaluations of the repaired cartilage. We recognize that articular cartilage has remarkable functional properties and the biomechanical properties of repaired cartilage are important for functional cartilage restoration. Thus, cartilage compressive test and creep test will be carried out in the near future.

## 5. Conclusion

Subchondral bone migration is related to cartilage repair for critical size osteochondral defects. Furthermore, the subchondral bone remodeling proceeds in a “flow like” manner and repaired cartilage with tidemark implies that the biphasic PEG/*β*-TCP composites fabricated by 3D printing provides a feasible strategy for osteochondral tissue engineering application.

## Supplementary Material

Wayne scoring system is modified cartilage assessment system of the Visual Histological Scale of the International Cartilage Repair Society(ICRS). The scale is composed of gross appearance and histology scores. For gross score evaluation, four items were involved including defect coverage, tissue color, defect margin, surface, with a total score of 16 points. Histology score is composed of matrix points, cell distribution points, smoothness points of the surface, Safranin O stain points, and Safranin O-stained area points, with a full histological score of 19 points.

## Figures and Tables

**Table 1:** Time dependent subchondral bone migration phenomenon during osteochondral repair period.

		Time points	
		Regression coefficients	*P* value
Subchondral bone volume	Linear *r* ^2^	0.638	<0.001
	Quadratic *r* ^2^	0.731	<0.001
	Cubic *r* ^2^	0.796	<0.001

Subchondral bone migration area	Linear *r* ^2^	0.639	<0.001
	Quadratic *r* ^2^	0.761	<0.001
	Cubic *r* ^2^	0.800	<0.001

**Table 2:** Correlation between repaired cartilage and subchondral bone migration.

	Subchondral bone volume		Subchondral bone migration area	
	Pearson's *r*	*P* value	Pearson's *r*	*P* value
Weeks	0.799	<0.001	0.799	<0.001
Gross appearance	0.865	0.001	0.923	<0.001
Histology	0.649	0.059	0.520	0.152
