# Supplementary figures and images for: Cartilage Repair and Subchondral Bone Migration Using 3D Printing Osteochondral Composites: A One-Year-Period Study in Rabbit Trochlea
Source: Biomed Res Int. 2014 Aug 7;2014:746138. doi: 10.1155/2014/746138 (PMC4142181; doi:10.1155/2014/746138)

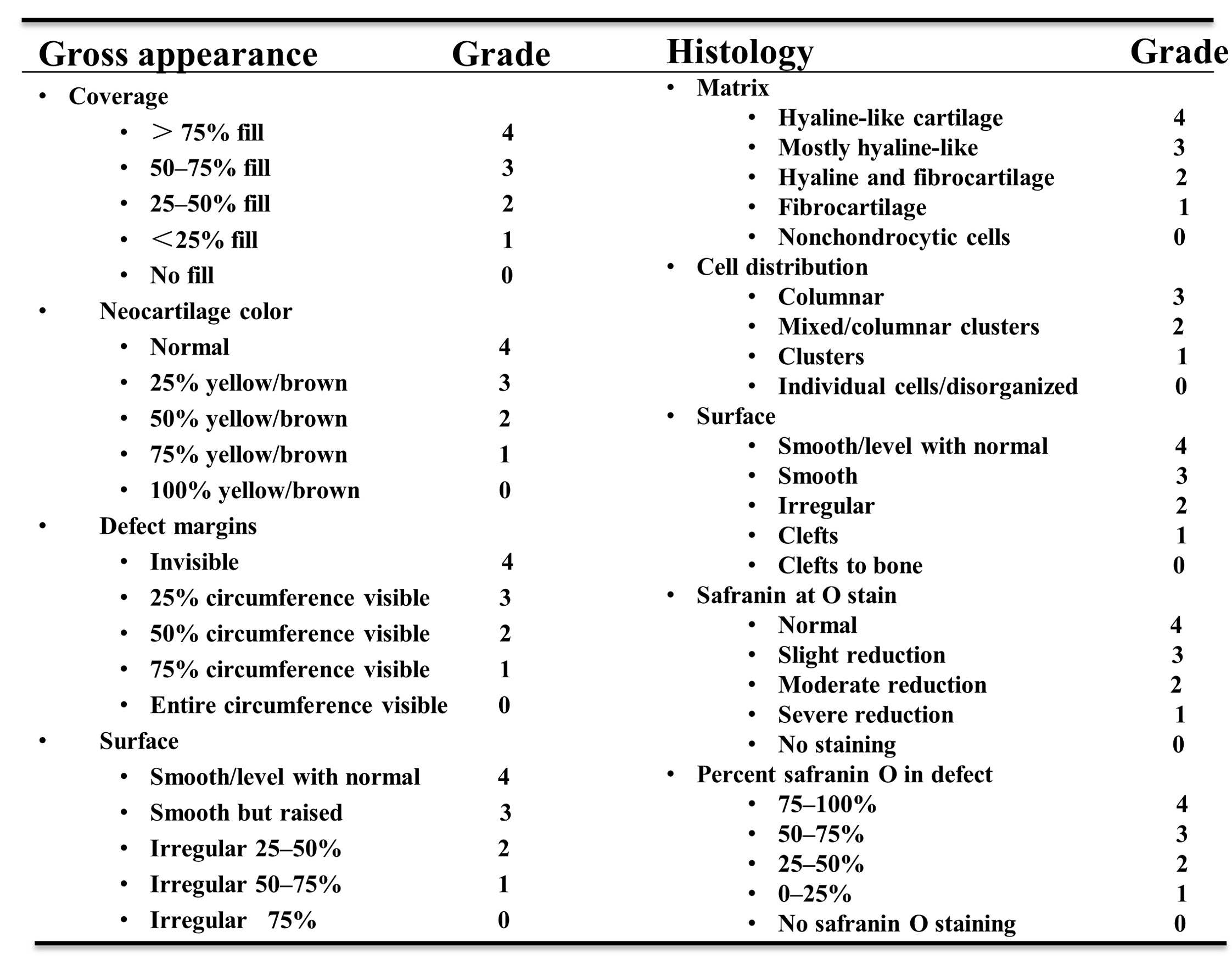

Supplement: Supplementary file 1 — Wayne scoring system is modified cartilage assessment system of the Visual Histological Scale of the International Cartilage Repair Society(ICRS). The scale is composed of gross appearance and histology scores. For gross score evaluation, four items were involved including defect coverage, tissue color, defect margin, surface, with a total score of 16 points. Histology score is composed of matrix points, cell distribution points, smoothness points of the surface, Safranin O stain points, and Safranin O-stained area points, with a full histological score of 19 points. [file 746138.f1.jpg]
